# Gender differences in tuberculosis incidence rates—A pooled analysis of data from seven high-income countries by age group and time period

**DOI:** 10.3389/fpubh.2022.997025

**Published:** 2023-01-10

**Authors:** Victoria Peer, Naama Schwartz, Manfred S. Green

**Affiliations:** School of Public Health, University of Haifa, Haifa, Israel

**Keywords:** tuberculosis, sex differences, gender, meta-analysis, meta-regression, male-to-female, incidence rate ratio

## Abstract

**Introduction:**

Gender differences in the incidence rates for tuberculosis are occasionally reported. However, the magnitude and consistency of the differences by age group, among different populations, and over extended periods of time are not clear.

**Materials and methods:**

We obtained national data from seven countries from open-access internet sites or personal communications with official representatives. We computed the male-to-female incidence rate ratios (IRRs) by country and year for every age group and pooled these ratios using meta-analytic methods. Meta-regression analysis was performed to estimate the contribution of age, country, and calendar years to the variation in the IRRs.

**Results:**

In the age groups of < 1, 1–4, 5–9, and 10–14, the pooled male-to-female IRRs (with 95% CI) were as follows: 1.21 (1.05, 1.40), 0.99 (0.95, 1.04), 1.01 (0.96, 1.06), and 0.83 (0.77, 0.89), respectively. In the age groups 15–44, 45–64, and 65+ years, incidence rates were significantly higher in men, with IRRs of 1.25 (1.16, 1.35), 1.79 (1.56, 2.06), and 1.81 (1.66, 1.96), respectively. Meta-regression analysis revealed that age significantly contributed to the variation in the IRRs.

**Conclusions:**

There were gender differences in the incidence rates for tuberculosis, with higher rates in boys aged less than one, no significant differences in boys of ages 1–9, and higher rates in boys/men older than 15. The only excess in female gender was in the age group 10–14 years. The age-related gender differences in tuberculosis incidence rates observed over several countries indicate the importance of including sex as a biological variable when assessing the risk factors for tuberculosis.

## Introduction

Tuberculosis (TB) is a significant cause of morbidity and mortality and is one of the leading causes of death due to a single infectious agent (1).

There is evidence of gender differences in the incidence of the disease, but it is not clear whether these differences are consistent over different age groups (2–10). Most of the reports on sex differences in tuberculosis incidence are based on specific subgroups or single countries. For example, in South India, the crude male-to-female TB incidence ratio was 2.4:1 (2).

In a population of Korean civil servants, the overall TB incidence rates were higher in men (240/100,000) than in women (163/100,000) in the age group 20–24 years (3). Most national TB studies in high-burden countries have reported a higher burden of disease among men, with male-to-female ratios of 1.2 in Ethiopia and 4.5 in Vietnam (4).

The existing information regarding sexual dimorphism in TB in high-income countries is controversial (5–10). In a study in Brazil (10), higher rates of infection were reported in girls aged 5–14, whereas in ages 15–39 years, they were higher in boys/men. In Germany (11) and France (12), overall tuberculosis incidence rates have been higher in boys/men.

To the best of our knowledge, gender differences in tuberculosis incidence rates by age groups, over different populations, and for prolonged periods of time have not been well-documented. In this study, we examine the type and magnitude of gender differences in tuberculosis incidence rates in high-income countries.

## Materials and methods

### Source of data

Sex-disaggregated TB incidence rates by age group are frequently difficult to access at the national level. We searched the official country sources from Europe, the American continent, and Australasia, in which reporting of tuberculosis by age and sex is mandatory and available in seven identified countries: England, Finland, Germany, Spain, Australia, Canada, and Israel. Data for Australia for 2001–2016 were obtained from the National Notifiable Diseases Surveillance System (NNDSS) (13), for Canada for 1991–2015 from the Canadian Notifiable Disease Surveillance System (CNDSS) (14), for England for 1990–2016 directly from Public Health England (PHE) representative, for Finland from the National Institute for Health and Welfare (THL) (15), for Germany for 2000–2015 from the German Federal Health Monitoring System (16), for Israel for 1998–2016 from the Department of Epidemiology in the Ministry of Health, and for Spain for 2005–2015 from the Spanish Epidemiological Surveillance Network (17).

Information about the population disaggregated by age, sex, and year for Australia was obtained from the Australian Bureau of Statistics (18), for Canada from Statistics Canada (19), for England from the Office for National Statistics (20), for Finland from the Statistics Finland's PX-Web databases (21), for Germany from the German Federal Health Monitoring System (22), for Israel from the Central Bureau of Statistics (23), and for Spain from the Demographic Statistics Database (24).

### Statistical analyses

For each country, we divided the entire study period, between the years 1990 and 2016, into 2–4 year-group intervals for a clearer presentation of the forest plots. We calculated incidence rates per 100,000 population (number of reported cases divided by the population size by sex and age group), for each country, between the years 1990 and 2016 for each year group separately. The male-to-female IRR was calculated by dividing the male incidence rate by that of the female incidence rate, according to age group, country of residency, and year group. The population was divided into seven age groups: < 1 (infants), 1–4 (early childhood), 5–9 (late childhood), 10–14 (puberty), 15–44 or 15–39 (young adulthood), 45–64 or 40–59 (middle adulthood), and 65+/60+ (older adulthood). The information reported from Canada, England, and Finland refers to the same age group as other countries except for the following: 15–39, 40–59, and 60 and beyond. Australia and Finland do not report disaggregated data for infants and children aged 1–4 years separately and were excluded from the analyses in those age groups.

As in our previous studies (25–28), we used meta-analytic statistical methodology to pool the IRRs over groups of years and countries. The meta-analyses were carried out using STATA software version 12.1 (Stata Corp., College Station, TX). The outcome variable was the male-to-female IRR. Overall IRRs for each age group were computed by combining the data (for all countries and year groups). Cochran's Q statistic was used for heterogeneity estimation. The variation between year groups for each country and age group was evaluated by using the Tau^2^ and *I*^2^. If *I*^2^ ≥ 50% and/or the Q test *p* < 0.1 (significant heterogeneity), the random effects model was performed to calculate overall IRRs and 95% confidence intervals (CI). Otherwise, the fixed effects model was applied. In this study, the power of the tests for heterogeneity was low, and we used the more conservative random effects model.

Sensitivity analysis was performed to detect the impact of the particular country or year groups on IRR, and the overall IRRs were computed after omitting one country or year group at a time. The impact of a particular country, year, or age on the heterogeneity of the overall IRRs was estimated using meta-regression.

## Results

**Figure 1:**
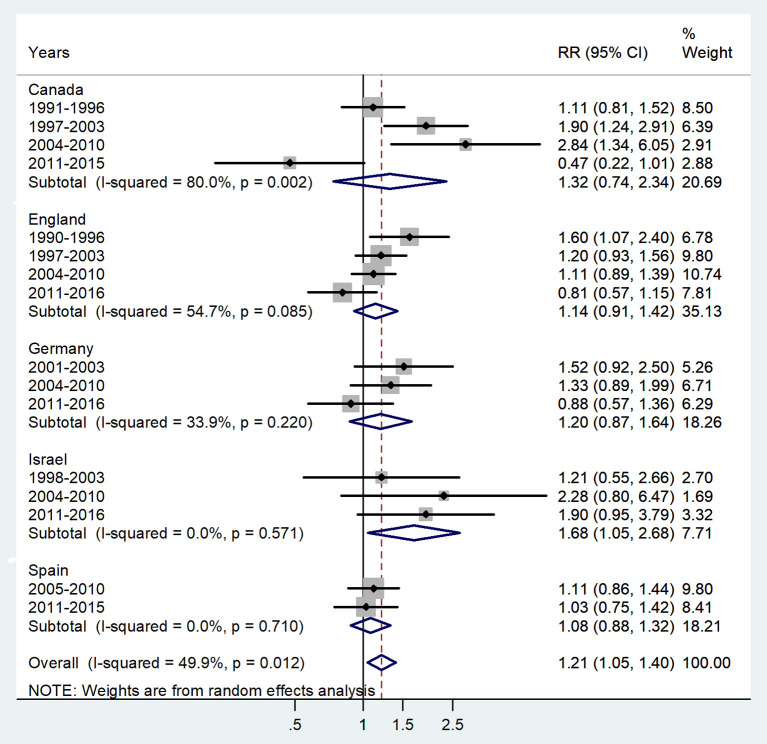
Forest plot of the male-to-female TB incidence RRs in infants for five countries by year groups.

**Figure 2:**
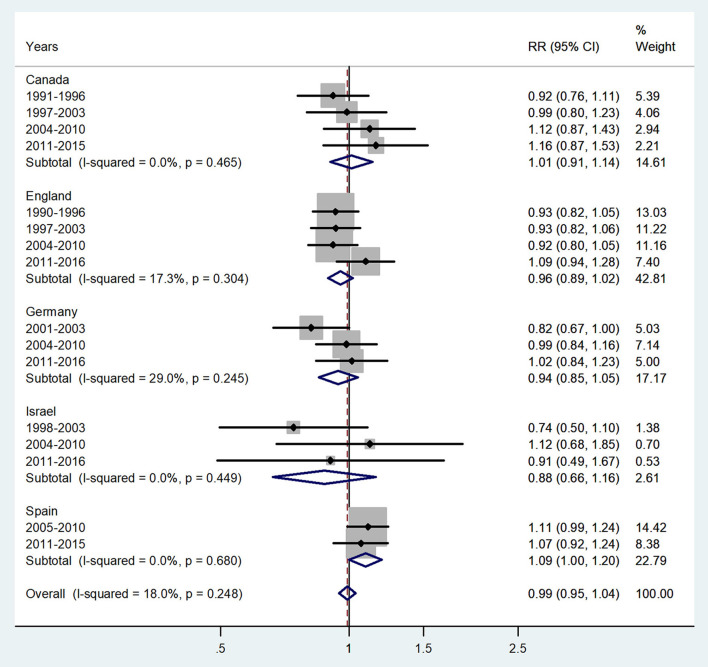
Forest plot of the male-to-female TB incidence RRs at ages 1–4 years for five countries by year groups.

**Figure 3:**
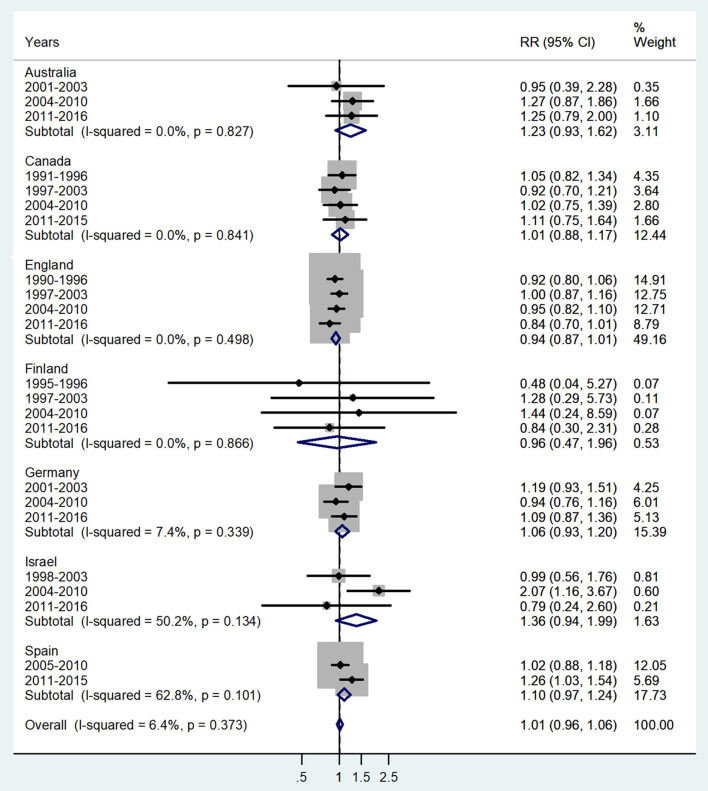
Forest plot of the male-to-female TB incidence RRs at ages 5–9 for seven countries by year groups.

**Figure 4:**
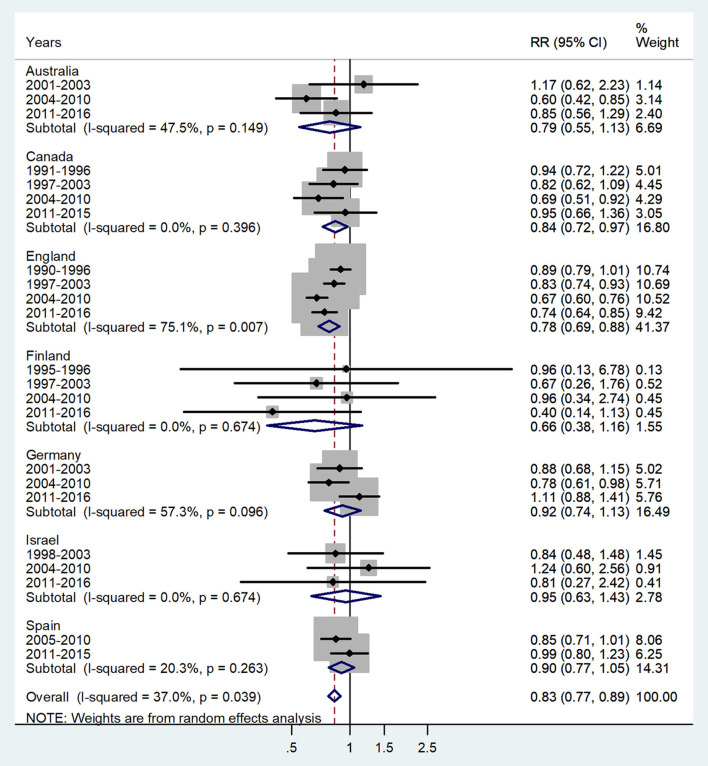
Forest plot of the male-to-female TB incidence RRs at ages 10–14 years for seven countries by year groups.

**Figure 5:**
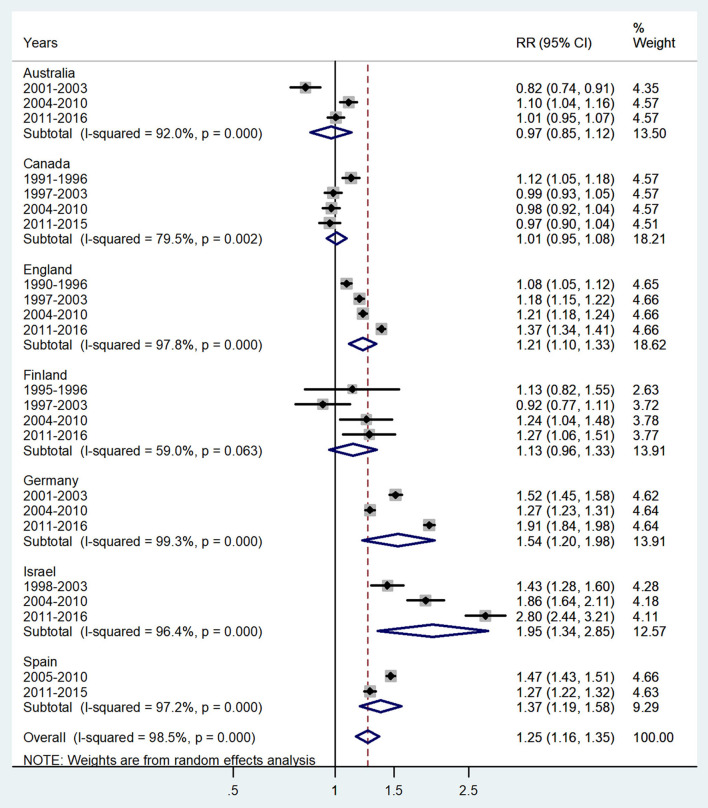
Forest plot of the male-to-female TB incidence RRs at ages 15–44 or 15–39 years for seven countries by year groups.

**Figure 6:**
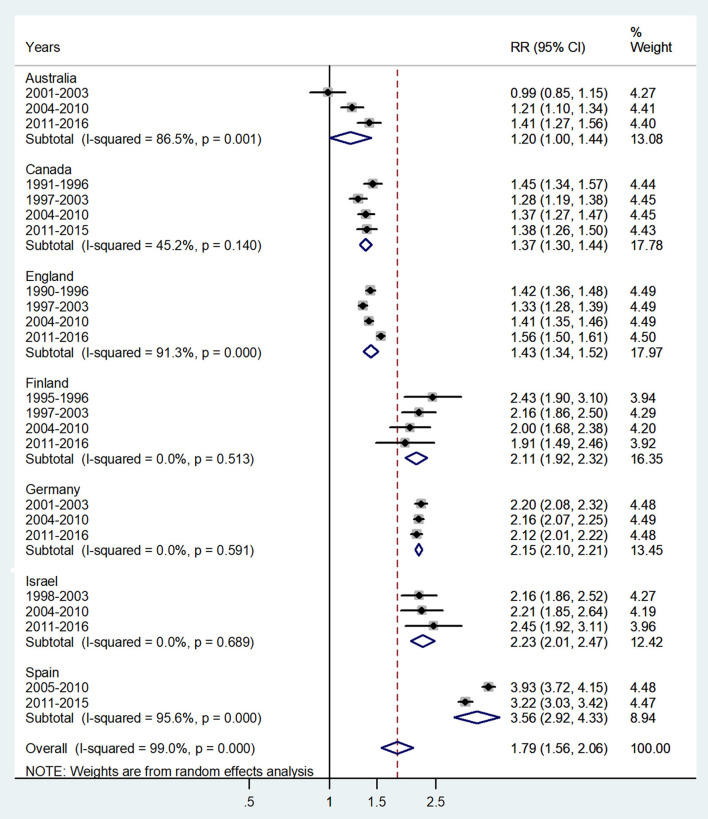
Forest plot of the male-to-female TB incidence RRs at ages 45–64 or 40–59 years for seven countries by year groups.

**Figure 7:**
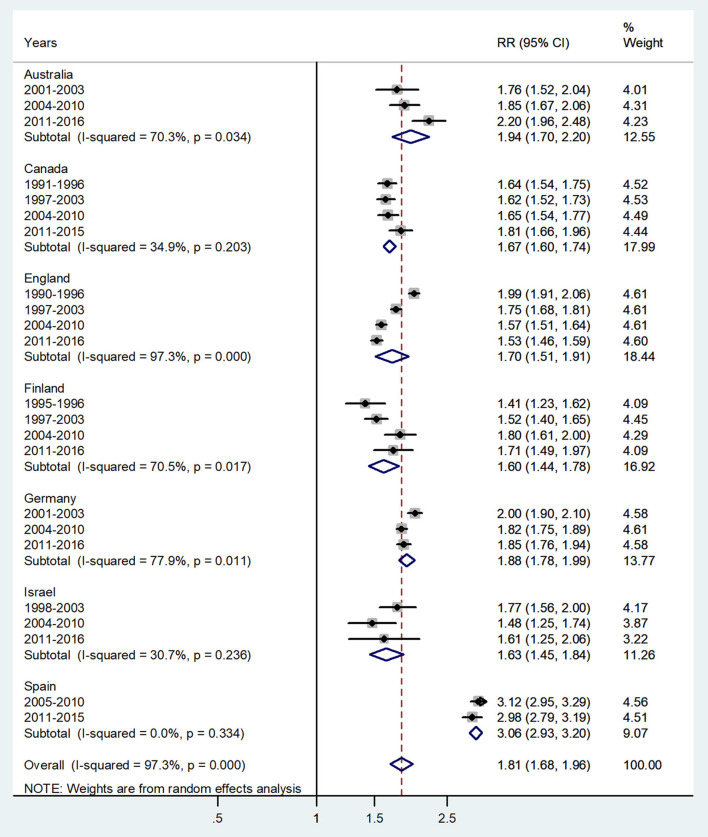
Forest plot of the male-to-female TB incidence RRs at age 60 and above or age 65 and above for seven countries by year groups. CI, 95% confidence interval.

The tuberculosis incidence rates (per 100,000 population) by gender, for each country, for different age groups and years are presented in Supplementary Table S1. The incidence rates between the countries varied widely. The highest incidence rate in both sexes in all age groups, except age 65 and beyond, was observed in England and Spain. The results of the pooled analyses by age group are presented as forest plots (Figures 1–7).

The forest plot for age < 1 year is presented in Figure 1. The overall male-to-female IRR in infants showed significant male preponderance with an IRR = 1.21 (95% CI 1.05–1.4), *p* = 0.012, and low heterogeneity (*I*^2^ = 49.9%). The IRRs varied from 1.08 in Spain to 1.68 in Israel.

The forest plot for ages 1–4 years is presented in Figure 2. No significant sex difference was evident, with an overall IRR = 0.99 (95% CI 0.95–1.04), *p* = 0.248 with *I*^2^=18%, and subtotal of IRRs varied from 0.88 in Israel to 1.09 in Spain.

The forest plot for ages 5–9 is given in Figure 3. No significant sex difference was observed with a pooled IRR = 1.01 (95% CI 0.96–1.06), *p* = 0.373, and *I*^2^ = 6.4%, and subtotal of IRRs by country varied from 0.94 in England to 1.36 in Israel.

The forest plot for ages 10–14 is presented in Figure 4. The overall male-to-female IRR in puberty showed significant female preponderance with a male-to-female IRR = 0.83 (95% CI 0.77–0.89), *p* = 0.039, and low heterogeneity (*I*^2^ = 37.0%). The subtotal of IRRs by country varied from 0.66 in Finland to 0.95 in Israel.

The forest plot for young adults (15–44 or 15–39 years) is shown in Figure 5. The overall male-to-female IRR in these ages showed higher incidence rates in boys with IRR = 1.25 (95% CI 1.16–1.35), *p* < 0.0001, and high heterogeneity (*I*^2^ = 98.5%). The subtotal of IRRs by country varied from 0.97 in Australia to 1.95 in Israel.

The forest plot for middle adulthood (45–64 or 40–59 years) is shown in Figure 6. The overall male-to-female IRR in these ages showed significantly higher incidence rates in male adults with IRR = 1.79 (95% CI 1.56–2.06), *p* < 0.0001, and high heterogeneity (*I*^2^ = 99.0%). The subtotal of IRRs by country varied from 1.20 in Australia to 3.56 in Spain.

The forest plot for ages 65 and above is given in Figure 7. The overall male-to-female IRR in these ages revealed significantly higher incidence rates in men with IRR = 1.81 (95% CI 1.68–0.96), *p* < 0.0001, and high heterogeneity (*I*^2^ = 97.3%). The subtotal of IRRs by country varied from 1.60 in Finland to 3.06 in Spain.

To identify the variables (particular country and year groups) that may have a significant impact on the overall IRR, we performed a leave-one-out sensitivity meta-analysis. After excluding one country or one year group at a time, the overall IRRs did not change significantly (Tables 1, 2, respectively). Egger's test for asymmetry was not significant for all age groups. Data are presented in Supplementary Figures S1A–G.

**Table 1:** Sensitivity analysis by country (after excluding one country at a time).

**Sensitivity by country**							
**Removed**	**Infants**	**Early**	**Late**	**Puberty**	**Young**	**Middle**	**Senior**
		**childhood**	**childhood**		**adulthood**	**adulthood**	**adulthood**
Australia	–	–	1.03 (0.95–1.11)	0.82 (0.78–0.86)	1.34 (1.18–1.53)	2.04 (1.5–2.76)	1.85 (1.57–2.18)
Canada	1.15 (1.04–1.29)	0.99 (0.94–1.04)	1.06 (0.95–1.17)	0.82 (0.78–0.86)	1.34 (1.18–1.52)	2.01 (1.45–2.77)	1.9 (1.59–2.26)
England	1.23 (1.06–1.42)	1.02 (0.96–1.08)	1.08 (1.01–1.16)	0.87 (0.81–0.94)	1.3 (1.09–1.54)	1.99 (1.43–2.77)	1.89 (1.55–2.3)
Finland	–	–	1.05 (0.96–1.14)	0.82 (0.78–0.86)	1.31 (1.15–1.5)	1.86 (1.38–2.52)	1.92 (1.63–2.26)
Germany	1.19 (1.05–1.36)	1 (0.95–1.05)	1.04 (0.94–1.16)	0.81 (0.77–0.85)	1.25 (1.1–1.41)	1.86 (1.31–2.63)	1.86 (1.53–2.27)
Israel	1.16 (1.06–1.28)	0.99 (0.95–1.04)	1.03 (0.95–1.11)	0.82 (0.78–0.86)	1.21 (1.06–1.38)	1.85 (1.37–2.5)	1.9 (1.62–2.23)
Spain	1.22 (1.08–1.37)	0.96 (0.91–1.01)	1.03 (0.94–1.13)	0.81 (0.77–0.85)	1.27 (1.09–1.48)	1.7 (1.39–2.08)	1.71 (1.62–1.81)

**Table 2:** Sensitivity analysis by years (after excluding group of years at a time).

**Removed**	**Infants**	**Early childhood**	**Late childhood**	**Puberty**	**Young adulthood**	**Middle adulthood**	**Senior adulthood**
**Sensitivity by years**							
1990–1996	1.16 (0.94–1.43)	1 (0.91–1.1)	1.02 (0.97–1.08)	0.8 (0.72–0.89)	1.29 (1.19–1.41)	1.78 (1.57–2.02)	1.83 (1.75–1.91)
1997–2003	1.13 (0.93–1.36)	1.01 (0.94–1.09)	1 (0.95–1.07)	0.82 (0.72–0.94)	1.25 (1.11–1.42)	1.74 (1.5–2.02)	1.87 (1.84–1.9)
2004–2010	1.18 (0.91–1.51)	0.97 (0.87–1.08)	1.01 (0.94–1.07)	0.86 (0.82–0.92)	1.23 (1.06–1.43)	1.62 (1.38–1.89)	1.82 (1.75–1.9)
2011–2016	1.27 (1.14–1.42)	0.96 (0.88–1.04)	1 (0.94–1.06)	0.82 (0.72–0.93)	1.19 (1.09–1.29)	1.63 (1.36–1.96)	1.83 (1.74–1.91)

Meta-regression analyses revealed that age group was responsible for much of the variation in IRRs. For infants, the male IRR was higher than that in puberty (*p* = 0.008), middle, and older adulthood (*p* < 0.0001). The IRRs for early and late childhood were significantly lower than that in the older age groups (*p* < 0.0001). Similarly, the IRR for puberty was significantly lower than in the older age groups (*p* < 0.0001).

## Discussion

In this study, we found higher tuberculosis incidence rates in boys/men at ages < 1, 15–44, 45–64, and 65 and above. At ages 10–14 years, we found a significant excess in girls. In this study of seven countries, in all age groups and both sexes, higher tuberculosis incidence rates were reported in England and Spain. This may reflect high incidence rates among immigrants to these countries. In 2014, for example, 6,520 cases were reported in England, with the highest incidence rate in London (29). In Spain, between 2005 and 2009, pediatric tuberculosis rates showed a slightly increasing tendency (30). The overall incidence of the disease in Spain remains higher than in many other high-income countries, especially among immigrants (31, 32).

We used the reported national population data to compute the TB IRRs from seven high-income countries with similar lifestyles, socioeconomic status, norms, and healthcare systems with equal access to healthcare for boys/men and girls/women. The study's findings may not apply equally to low-income countries. Thus, the data should be largely representative of the high-income countries of Europe, North America, Australia, and Israel and cannot be generalized at a global level.

Gender differences in tuberculosis incidence rates are frequently mentioned, but quantitative estimates of the differences in large populations, especially in developed countries, are poorly documented. All countries contributed large populations and numbers of cases over many years. A possible source of selection bias could be gender-biased medical care-seeking patterns or immunization rates in childhood. To the best of our knowledge, there is no evidence of such a bias in any of the countries in this study. Information bias could be present due to under-reporting or underdiagnosis. However, such a bias should not differ between genders. This could lead to non-differential misclassification bias, which could result in an underestimation of the gender differences in incidence rates. In addition, the sensitivity of the diagnosis of tuberculosis could vary between countries and over time periods but should not differ between sexes and thus be non-differential. One of the reasons for the potential bias could be the inability to match the TB incidence data to HIV registries.

Possible sources of bias could be different rates of homelessness and immigration patterns. The gender-related differences in homelessness are under-researched because of a lack of data on the nature and extent of women's homelessness. Across Europe, homelessness among women was defined as a minor social problem in comparison to overall homelessness which is experienced by adult men. This interpretation of homelessness in Europe is a misconception because of hidden forms of homelessness among women (33, 34).

The information about gender-related immigration is also controversial. The proportion of female immigrants in the world increased from 47.9% to 50% in 2005. In Europe, women accounted for 52.4% of the migrants in 2000 (35). In contrast, according to other sources, the demographic profile of asylum seekers in Europe has become more male predominant (36).

Overall, our results are consistent with the few national studies in individual high-income countries, with higher incidence rates reported among boys/men (5, 7). A study conducted in Hong Kong found that tuberculosis rates were higher in men than in women across all age groups (5). In San Francisco, until the age of 24, the incidence of tuberculosis in boys/men and girls/women was similar. After age 25, the incidence in men was two to three times higher than that in women (6). Among TB patients in Victoria from 2002 to 2015, in all age groups, the male-to-female ratio was 1.2 (7). In 2016, in Germany, the age-specific incidence was highest in the age group of 20–24 years (18.3 cases per 100,000 population; 25.9 in men and 9.7 in women) (8). Between 1999 and 2006, there were 1,370 cases of TB in children in London with a higher incidence in girls under 15 years (37). The study (10) conducted in Vitoria, Brazil, found that the increase in TB infection was significant in girls aged 5–14 years and in boys/men aged 15–39 years. Sex differences disappeared by age 40 (10). In contrast, in a study in Tuscany, no significant male-to-female differences in tuberculosis incidence were found among children aged 0–14 years (9).

The reasons for higher incidence in boys/men in almost all age groups are not clear.

### Behavioral, socioeconomic differences, and risk factors

#### Adult groups

The observed gender and age-related differences in tuberculosis incidence in adults (age 15–44, 45–64, and 65 and beyond) may be explained, at least in part, by behavioral and cultural differences. Social contact patterns contribute to the excess burden of tuberculosis in men. Differences in gender- and age-specific social contact likely contribute to sex disparities in adult tuberculosis burden by increasing incidence among men (38).

Other risk factors such as smoking, alcohol consumption, poor nutrition, and HIV comorbidity increase the susceptibility to tuberculosis (39). Risk factors such as alcohol consumption and smoking are usually more prevalent among men than among women, and researchers established a correlation between these elements and the risk of developing TB (39). Theoretically, malnutrition and the differences in the balance between the physiological need for basic nutrients and the gender-biased access to these nutrients provided by society could explain, in part, excess TB incidence in men, but it is unlikely to be the explanation for gender-related bias in high-income countries (40).

One of the significant risk factors predisposing an individual to TB incidence is HIV comorbidity (41). Immigrants (most of them are men; they are the first to arrive in a country and are more likely to work in occupations with higher potential exposures) from developing, low- and middle-income countries are the populations significantly affected by HIV than those from many high-income countries, such as countries in Europe, Canada, Australia, and New Zealand (42). Although the impact of HIV on female TB was much more significant, male TB rates remained higher (43, 44). This fact emphasizes that other factors besides HIV comorbidity influence TB incidence.

#### Efficacy of BCG vaccine

One of the possible explanations may be sex-related differences in the efficacy of the Bacillus Calmette–Guérin (BCG) vaccine. BCG immunization had been recommended by healthcare providers until the 1980s in Australia, Spain, and Israel when the universal vaccination against TB stopped (45). In other countries the vaccination stopped later: 1990 in New Zealand, 1998 in Germany, 2006 in Finland, and 2010 in the Czech Republic. Over time, there have been policy changes in England with respect to BCG vaccination, induced by changes in the epidemiology of TB (46).

The duration of protection from tuberculosis morbidity of BCG vaccines is not clear. In a study on American Indians and Alaska Natives, there was a slight waning of the efficacy of BCG vaccination over time, higher among men than women (47). In Guinea-Bissau, the long-term protective effects of the BCG vaccine on overall survival and reduced susceptibility to respiratory infections were greater for girls than boys (48, 49).

Sex hormones could modulate BCG-induced effects in infancy as well as in adults. However, different mechanisms are operating in these different age groups. Koeken et al. (50) found a negative association between baseline testosterone concentrations and changes in circulating proteins after BCG vaccination in men, suggesting a possible role for testosterone in sex-differential effects after BCG vaccination.

#### Migrants

In high-income countries, migration from countries with a high burden of tuberculosis influences the local epidemiology and is responsible for the increase in disease incidence (51). The decrease in the number of TB cases among the native population in European countries is sometimes accompanied by an increase in TB cases among foreign immigrants (52).

#### Underdiagnosis

Hypothetically, such differences in TB incidence between sexes may be explained by under-diagnosis or under-reporting of tuberculosis by girls/women or by real epidemiological differences in infection with Mycobacterium tuberculosis (53). Observed gender-based differences in TB rates between male and female adults are due to differences in transmission dynamics rather than underdiagnosis and under-reporting in female adults (6).

No facts were found to support the assumption that male adults were slower to access care or were less well served by healthcare services (7).

The immune and inflammatory status of an adult individual can be influenced by environmental factors, age, and sex. Multiple signaling immune system-related pathways can be modified by sex hormones and impact the innate immune response in one direction or the other. To what extent sex differences in immunity could contribute to the adult male bias in TB morbidity remains unclear (54).

### Infancy and puberty

#### Biological factors?

Factors such as exposure, behavior, and co-morbidities are less likely to impact gender differences in tuberculosis incidence in infancy. This could be explained, at least in part, by biological and physiological factors influencing the immune response to the pathogen (55). Multiple fluctuations in hormonal status that occur during infancy and puberty may affect the activity of the immune system. Mini-puberty occurs in infants of both sexes, with an increase in testosterone in boys and an increase in both estrogen and testosterone in girls. These hormonal changes may impact the pathogenesis of TB (56). To the best of our knowledge, in children and especially in children of pubertal age, there are no data to suggest that girls have more frequent exposure to infectious TB cases compared to boys of the same age (57). Nevertheless, for reasons unknown, the reactivation of tuberculosis among adolescents affects girls two times as often as boys (55). At puberty, there is a female preponderance in disease incidence. Similar findings have been published in other studies (10, 37). Girls are more exposed to infection in the household than boys, perhaps because they are more active within the home. Another possible explanation may be sex-related physiological differences in susceptibility to TB. During puberty, sex hormone levels fluctuate significantly (58). Estrogen plays a protective role against infection, increasing the immune response, cytokine, and macrophage activity (59, 60). It is plausible that since girls aged 10–14 years are partly pre-pubertal, the protective hormonal effect is not significant enough, making them more susceptible to TB. Compared to girls, boys have a stronger Th1 profile and increased numbers of CD8+ T cells and NK cells. Girls, therefore, elicit more enhanced inflammatory responses (61).

In summary, it is plausible that, in adulthood, behavioral and socioeconomic factors play a significant role in gender differences in TB incidence. These factors are clearly important, but it is difficult to evaluate their contribution to gender-related TB incidence. We suggest that sex hormones, both during infancy and in puberty, cause functional differences between male and female immune cells. These are involved in part in the host-pathogen interaction and possible impact on the outcome of TB infection. To what extent these sex differences contribute to gender bias in TB incidence remains insufficiently understood and requires further study.

## Conclusions

The sex differences in the incidence of TB are not always addressed in interventions and research. Addressing the excess burden of TB in adult men is essential to improve men's health and to meet the ambitious targets for reducing TB incidence and deaths. Sex differences in children imply that there is still much about TB that we do not understand. Immunological changes in infants and puberty are likely to have a central role in the response to TB and require further research to understand the underlying biological mechanisms. A better understanding of the sex and gender-related immunological basis for the male excess in disease should encourage the development of personalized therapies. New vaccines in development should be tested for their efficacy in boys/men and girls/women separately. Public health strategies for TB eradication and prevention must be designed according to age and gender.

## Data availability statement

The original contributions presented in the study are included in the article/Supplementary material, further inquiries can be directed to the corresponding author.

## Ethics statement

Ethical review and approval was not required for the study on human participants in accordance with the local legislation and institutional requirements. Written informed consent from the participants' legal guardian/next of kin was not required to participate in this study in accordance with the national legislation and the institutional requirements.

## Author contributions

VP participated in the study design, collected the data, helped in the interpretation of the analyses, and writing the manuscript. MG designed and supervised the study, participated in the analyses and interpretation of the data, and in writing the manuscript. NS assisted in the data analysis and contributed important aspects to the review of the manuscript. All authors approved the final version submitted.
