# Corrigendum: Gender differences in tuberculosis incidence rates—A pooled analysis of data from seven high-income countries by age group and time period

**DOI:** 10.3389/fpubh.2023.1157235

**Published:** 2023-02-21

**Authors:** Victoria Peer, Naama Schwartz, Manfred S. Green

**Affiliations:** School of Public Health, University of Haifa, Haifa, Israel

**Keywords:** tuberculosis, sex differences, gender, meta-analysis, meta-regression, male-to-female, incidence rate ratio

In the published article, there was an error in Table 2 as published. The same Table 2, Sensitivity analysis by years (after excluding group of years at a time), appears twice as a duplicate table in the “Results” section. The corrected Table 2 and its caption appear below.

**Table 2:** Sensitivity analysis by years (after excluding group of years at a time).

**Removed**	**Infants**	**Early childhood**	**Late childhood**	**Puberty**	**Young adulthood**	**Middle adulthood**	**Senior adulthood**
**Sensitivity by years**							
1990–1996	1.16 (0.94–1.43)	1 (0.91–1.1)	1.02 (0.97–1.08)	0.8 (0.72–0.89)	1.29 (1.19–1.41)	1.78 (1.57–2.02)	1.83 (1.75–1.91)
1997–2003	1.13 (0.93–1.36)	1.01 (0.94–1.09)	1 (0.95–1.07)	0.82 (0.72–0.94)	1.25 (1.11–1.42)	1.74 (1.5–2.02)	1.87 (1.84–1.9)
2004–2010	1.18 (0.91–1.51)	0.97 (0.87–1.08)	1.01 (0.94–1.07)	0.86 (0.82–0.92)	1.23 (1.06–1.43)	1.62 (1.38–1.89)	1.82 (1.75–1.9)
2011–2016	1.27 (1.14–1.42)	0.96 (0.88–1.04)	1 (0.94–1.06)	0.82 (0.72–0.93)	1.19 (1.09–1.29)	1.63 (1.36–1.96)	1.83 (1.74–1.91)

The authors apologize for this error and state that this does not change the scientific conclusions of the article in any way. The original article has been updated.

