# Supplementary figures and images for: Gender differences in tuberculosis incidence rates—A pooled analysis of data from seven high-income countries by age group and time period
Source: Front Public Health. 2023 Jan 10;10:997025. doi: 10.3389/fpubh.2022.997025 (PMC9873377; doi:10.3389/fpubh.2022.997025)

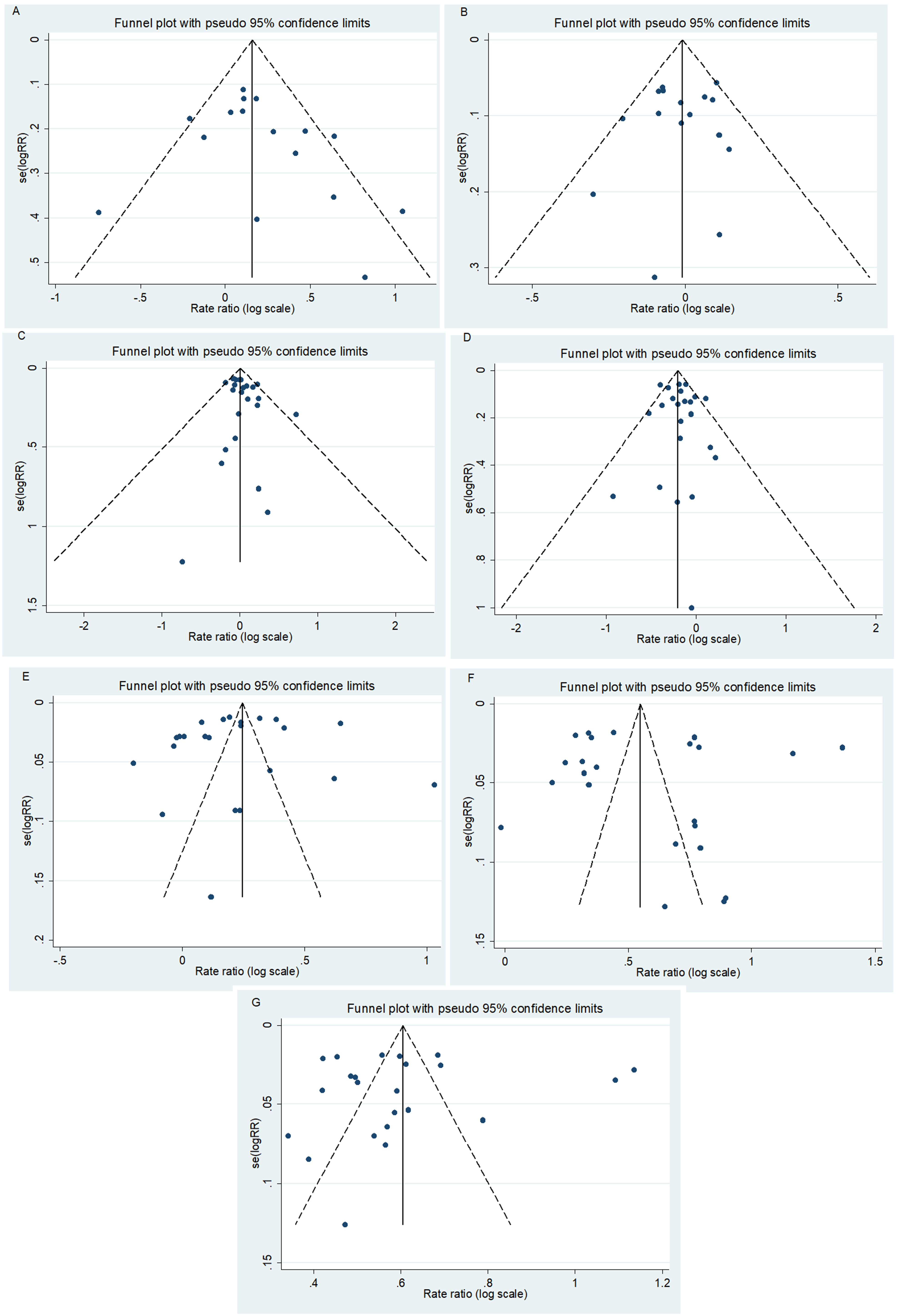

Supplement: Supplementary file 1 [file Image_1.TIF]
