# Supplementary material for: Gender differences in tuberculosis incidence rates—A pooled analysis of data from seven high-income countries by age group and time period
Source: Front Public Health. 2023 Jan 10;10:997025. doi: 10.3389/fpubh.2022.997025 (PMC9873377; doi:10.3389/fpubh.2022.997025)
Supplement: Supplementary file 2 [file Table_1.DOCX]

**Table S1: Data from all Countries Included in the Meta-Analysis, by Sex and Age Group - Descriptive Data**

| **Age** | **Country** | **Years** | **Male, n** | **Male, N** | **Female, n** | **Female, N** | **IR male** | **IR female** | **IRR** |
| --- | --- | --- | --- | --- | --- | --- | --- | --- | --- |
| **<1** | Canada | 1991-2015 | 185 | 4682619 | 133 | 4446799 | 3.95 | 2.99 | 1.32 |
|  | England | 1990-2016 | 426 | 8725051 | 358 | 8306732 | 4.88 | 4.31 | 1.13 |
|  | Germany | 2001-2016 | 136 | 5740478 | 108 | 5448550 | 2.37 | 1.98 | 1.20 |
|  | Israel | 1998-2016 | 50 | 1486100 | 28 | 1410400 | 3.36 | 1.99 | 1.69 |
|  | Spain | 2005-2015 | 205 | 2679186 | 178 | 2514548 | 7.65 | 7.08 | 1.08 |
| **1-4** | Canada | 1991-2015 | 620 | 19156418 | 582 | 18225737 | 3.24 | 3.19 | 1.01 |
|  | England | 1990-2016 | 1710 | 34821935 | 1707 | 33207057 | 4.91 | 5.14 | 0.96 |
|  | Germany | 2001-2016 | 680 | 23509315 | 683 | 22311030 | 2.89 | 3.06 | 0.94 |
|  | Israel | 1998-2016 | 96 | 5731500 | 104 | 5443300 | 1.67 | 1.91 | 0.88 |
|  | Spain | 2005-2015 | 1049 | 10880587 | 903 | 10233932 | 9.64 | 8.82 | 1.09 |
| **5-9** | Australia | 2001-2016 | 114 | 11398585 | 88 | 10814642 | 1.00 | 0.81 | 1.23 |
|  | Canada | 1991-2015 | 375 | 24668602 | 352 | 23469919 | 1.52 | 1.50 | 1.01 |
|  | England | 1990-2016 | 1366 | 42989082 | 1393 | 41012194 | 3.18 | 3.40 | 0.94 |
|  | Finland | 1995-2016 | 15 | 3440956 | 15 | 3297629 | 0.44 | 0.45 | 0.96 |
|  | Germany | 2001-2016 | 485 | 30760941 | 435 | 29187252 | 1.58 | 1.49 | 1.06 |
|  | Israel | 1998-2016 | 66 | 6616300 | 46 | 6287700 | 1.00 | 0.73 | 1.36 |
|  | Spain | 2005-2015 | 580 | 13017097 | 500 | 12287011 | 4.46 | 4.07 | 1.09 |
| **10-14** | Australia | 2001-2016 | 111 | 11377822 | 141 | 10797396 | 0.98 | 1.31 | 0.75 |
|  | Canada | 1991-2015 | 338 | 25685783 | 383 | 24391864 | 1.32 | 1.57 | 0.84 |
|  | England | 1990-2016 | 1815 | 42597565 | 2207 | 40624659 | 4.26 | 5.43 | 0.78 |
|  | Finland | 1995-2016 | 21 | 3522497 | 31 | 3375446 | 0.60 | 0.92 | 0.65 |
|  | Germany | 2001-2016 | 389 | 33455166 | 402 | 31724889 | 1.16 | 1.27 | 0.92 |
|  | Israel | 1998-2016 | 46 | 6106400 | 46 | 5807300 | 0.75 | 0.79 | 0.95 |
|  | Spain | 2005-2015 | 411 | 12301238 | 432 | 11627137 | 3.34 | 3.72 | 0.90 |
| **15-44/15-39** | Australia | 2001-2016 | 5706 | 73591102 | 5542 | 72741755 | 7.75 | 7.62 | 1.02 |
|  | Canada | 1991-2015 | 8619 | 143987472 | 8283 | 140453550 | 5.99 | 5.90 | 1.02 |
|  | England | 1990-2016 | 45921 | 234901126 | 37299 | 233399206 | 19.55 | 15.98 | 1.22 |
|  | Finland | 1995-2016 | 861 | 18898064 | 724 | 18050351 | 4.56 | 4.01 | 1.14 |
|  | Germany | 2001-2016 | 23149 | 257895408 | 14397 | 247590330 | 8.98 | 5.81 | 1.54 |
|  | Israel | 1998-2016 | 2238 | 29586200 | 1166 | 29264100 | 7.56 | 3.98 | 1.90 |
|  | Spain | 2005-2015 | 18686 | 110542308 | 12714 | 105413400 | 16.90 | 12.06 | 1.40 |
| **45-64/40-59** | Australia | 2001-2016 | 2105 | 41988401 | 1718 | 42573071 | 5.01 | 4.04 | 1.24 |
|  | Canada | 1991-2015 | 6185 | 110461323 | 4502 | 109655649 | 5.60 | 4.11 | 1.36 |
|  | England | 1990-2016 | 25322 | 175100277 | 17938 | 177644620 | 14.46 | 10.10 | 1.43 |
|  | Finland | 1995-2016 | 1360 | 16513241 | 635 | 16307550 | 8.24 | 3.89 | 2.12 |
|  | Germany | 2001-2016 | 15966 | 181698132 | 7415 | 181849520 | 8.79 | 4.08 | 2.15 |
|  | Israel | 1998-2016 | 1083 | 12368500 | 525 | 13327000 | 8.76 | 3.94 | 2.22 |
|  | Spain | 2005-2015 | 10427 | 63103755 | 2946 | 64340310 | 16.52 | 4.58 | 3.61 |
| **65+/60+** | Australia | 2001-2016 | 2098 | 21417772 | 1292 | 25538457 | 9.80 | 5.06 | 1.94 |
|  | Canada | 1991-2015 | 7565 | 64590224 | 5547 | 78346403 | 11.71 | 7.08 | 1.65 |
|  | England | 1990-2016 | 24140 | 129663953 | 17865 | 163257756 | 18.62 | 10.94 | 1.70 |
|  | Finland | 1995-2016 | 2842 | 11159619 | 2491 | 15066114 | 25.47 | 16.53 | 1.54 |
|  | Germany | 2001-2016 | 13447 | 108019284 | 10123 | 149862231 | 12.45 | 6.75 | 1.84 |
|  | Israel | 1998-2016 | 992 | 6010700 | 802 | 7903600 | 16.50 | 10.15 | 1.63 |
|  | Spain | 2005-2015 | 6766 | 37127234 | 2974 | 49879431 | 18.22 | 5.96 | 3.06 |
